# Direct Endovascular Thrombectomy or With Prior Intravenous Thrombolysis for Acute Ischemic Stroke: A Meta-Analysis

**DOI:** 10.3389/fneur.2021.752698

**Published:** 2021-12-13

**Authors:** Jing Chen, Teng-Fei Wan, Tian-Ce Xu, Guo-Can Chang, Hui-Sheng Chen, Liang Liu

**Affiliations:** ^1^Department of Neurology, Central Hospital of Baoji, Baoji, China; ^2^Department of Critical Care Medicine, The General Hospital of Northern Theater Command, Shenyang, China; ^3^Department of Neurology, The General Hospital of Northern Theater Command, Shenyang, China

**Keywords:** acute ischemic stroke, endovascular thrombectomy, intravenous thrombolysis, bridging thrombolysis, meta-analysis

## Abstract

**Background and purpose:** It is unclear whether endovascular thrombectomy alone compared with intravenous thrombolysis combination with endovascular thrombectomy can achieve similar neurological outcomes in patients with acute large vessel occlusion stroke. We aimed to perform a systematic review and meta-analysis of randomized controlled trials to compare endovascular thrombectomy alone or intravenous thrombolysis plus endovascular thrombectomy in this population.

**Methods:** We systematically searched PubMed, Embase, and ClinicalTrials.gov. We restricted our search to randomized clinical trials that examined the clinical outcomes of endovascular thrombectomy alone vs. intravenous thrombolysis plus endovascular thrombectomy. The Cochrane risk of bias tool was used to assess study quality. Random-effects meta-analyses were used for evaluating all outcomes.

**Results:** Total three randomized controlled trials with 1,092 individuals enrolled were included in the meta-analysis, including 543 (49.7%) who received endovascular thrombectomy alone and 549 (50.3%) who received intravenous thrombolysis plus endovascular thrombectomy. The primary outcome of 90-day functional independence (modified Rankin scale (mRS) score ≤ 2) was 44.6% (242/543) in the endovascular thrombectomy alone group vs. 42.8% (235/549) in the alteplase with endovascular thrombectomy group (odds ratio (OR), 1.08 [95% CI, 0.85–1.38]; *P* = 0.0539). Among pre-specified secondary outcomes, no significant between-group differences were found in excellent outcome (mRS score ≤ 1) (OR, 1.12 [95% CI, 0.85–1.47]; *P* = 0.418), mortality at 90 days (OR, 0.93 [95% CI, 0.68–1.29]; *P* = 0.673), successful reperfusion (thrombolysis in cerebral infarction 2b-3) (OR, 0.75 [95% CI, 0.54–1.05]; *P* = 0.099), and symptomatic intracranial hemorrhage (OR, 0.72 [95% CI, 0.45–1.15]; *P* = 0.171).

**Conclusions:** Among patients with acute ischemic stroke in the anterior circulation within 4.5 h from the onset, endovascular thrombectomy alone was non-inferior to combined intravenous thrombolysis and endovascular thrombectomy.

## Background

Endovascular thrombectomy (EVT) has become a standard treatment for acute ischemic stroke patients caused by a large vessel occlusion in the anterior circulation (1–3). A subsequent analysis of individual patient data from five randomized trials showed that the effect of EVT was not influenced by prior intravenous thrombolysis (IVT), raising the question of whether treatment with IVT before EVT is still necessary (3). A *post hoc* analysis of the Solitaire With the Intention for Thrombectomy (SWIFT) and Solitaire Flow Restoration Thrombectomy for Acute Revascularization (STAR) studies indicates that EVT combined with standard alteplase treatment does not appear to provide a clinical benefit over EVT alone (4). In contrast, a meta-analysis of 13 studies suggested a better functional outcome, lower mortality, and higher rate of successful recanalization in patients treated with EVT and bridging IVT (5). However, in these 13 studies, a substantial number of patients with stroke received EVT alone who are not eligible for IVT due to unknown onset of stroke symptoms or contraindications to IVT. The eligibility for IVT may lead to group imbalances in stroke etiology, risk factors, and time to treatment. Thus, to eliminate the confusion about the eligibility for IVT between groups, the benefit and risk of direct EVT vs. EVT with prior IVT should be determined for patients with stroke who are eligible for IVT. To test the hypothesis that EVT alone was non-inferior to combined IVT and EVT in patients with a large vessel occlusion in the anterior circulation treated within 4.5 h of onset, three recent large randomized controlled trials were conducted (6–8). In this study, we intended to conduct a meta-analysis including complete results from recently published randomized controlled trials to compare effectiveness and safety between direct EVT and bridging therapy (EVT with prior IVT) for acute ischemic stroke with large vessel occlusions. Both included patients in direct EVT and bridging therapy groups who had no contraindications to IVT. Our results may provide more pieces of evidence to develop best practice guidelines for patients with acute ischemic stroke with large vessel occlusions.

## Methods

This systematic review and meta-analysis was conducted using a pre-specified protocol following the Preferred Reporting Items for Systematic Reviews and Meta-Analyses (PRISMA) statement (9).

### Search Strategy and Inclusion Criteria

We searched PubMed, Embase, and the clinical trial registry maintained at ClinicalTrials.gov until April 16, 2021, using the terms “intravenous thrombolysis or intravenous alteplase” and “acute ischemic stroke or cerebrovascular ischemia” and “endovascular therapy or endovascular treatment or mechanical thrombectomy (MT) or stent-retriever.” The references of published reviews and studies with potential met our pre-specified inclusion and exclusion criteria were manually screened to avoid missing any eligible studies that were not previously identified. We restricted studies published in the English language. Two investigators (LL and TFW) independently conducted the literature search. To facilitate higher quality evidence, we used strict inclusion and exclusion criteria for each study. Inclusion criteria were the following: (1) compared outcomes of IVT + EVT with EVT in acute ischemic stroke of large artery occlusion primarily in anterior circulation; (2) all the participants could be treated with IVT within 4.5 h after symptom onset; (3) reported functional outcome using the modified Rankin scale (mRS) as an endpoint; (4) reported the effect estimates of studies or calculating the effect estimates from the available data; and (5) a randomized clinical trial study design. We excluded case reports, reviews, *post hoc* analyses, observational studies, duplicate reports, commentaries, abstracts, animal studies, meeting proceedings, and studies with incomplete information. Moreover, studies included patients who are not eligible for IVT due to unknown onset of stroke symptoms or contraindications to IVT, which were also excluded.

### Data Extraction and Outcomes

The study and patient characteristics, data on outcomes were abstracted by two authors (TFW and TCX) independently from article texts, tables, figures, supplementary appendixes, and protocols. Any disagreements were resolved by joint discussion. The study and patient characteristics were extracted including author name, publication year, study design type, study period, sources of data, inclusion and exclusion criteria, outcomes, and sample size in each group.

The primary outcome was three-month functional independence that was defined as a mRS score of 0–2. Secondary outcomes were the following: early recanalization and reperfusion thrombolysis in cerebral infarction (TICI) score 2b/3 after MT, symptomatic intracranial hemorrhage (sICH), asymptomatic intracranial hemorrhage (aICH), mortality at 90 days, 3-month favorable outcome (mRS 0–1).

### Quality Assessment

Quality assessment of the studies was performed by two independent reviewers (LL and TFW). The risk of bias for each included RCT was assessed according to the Cochrane Collaboration's tool (10), which includes each of the following domains: sequence generation, allocation concealment, blinding of participants, personnel and outcome assessors, incomplete outcome data, reporting biases, and other potential sources of bias. The risk of bias was assigned as a score of low, unclear, or high, according to established criteria. The study with more than two high-risk components, was defined as having a moderate risk of bias. And the study with more than four high-risk components, was defined as having a high risk of bias. While the study with 0–2 high-risk components was defined as having a low risk of bias.

### Statistics

From each study, we extracted a 2 × 2 table for binary outcomes. Meta-analysis results were expressed as odds ratios (ORs) for binary outcomes with respective 95% CIs. ORs with their 95% CIs were used as a measure of the association of EVT with each outcome of interest compared to IVT + EVT. The random-effects meta-analysis model (DerSimonian–Laird method) or fixed-effects meta-analysis model (Mantel–Haenszel method) was used to pool count data across trials and the statistical significance of pooled ORs and 95% CIs were determined with an equivalent *Z*-test (11). Which model should be used for pooling count data across trials was following the heterogeneity among the included RCTs. The heterogeneity among the RCTs included in our meta-analysis was assessed by the *P*-value of chi-squared-based *Q*-tests and the *I*-squared (*I*^2^) statistic. As the previous study reported, the *I*^2^ value was <50% and the *P*-value of the *Q*-test was more than 0.1 among the RCTs included in the meta-analysis, which may suggest no obvious heterogeneity across studies. Then the fixed-effects model was used for pooling across studies. While the *I*^2^ values of more than 50% and the *P*-value of the *Q*-test of <0.1 may indicate the studies included in the meta-analysis with obvious heterogeneity. Then the random-effects model was used (12). Statistical analyses were conducted using STATA software, version 12.0 (StataCorp, College Station, TX, USA). Statistical significance was set to *P* < 0.05.

## Results

### Study Selection and Study Characteristics

A total of three trials met the inclusion criteria and were included in this meta-analysis (online-only Data Supplement): Direct Intraarterial Thrombectomy in Order to Revascularize Acute Ischemic Stroke Patients with Large Vessel Occlusion Efficiently in Chinese Tertiary Hospitals: a Multicenter Randomized Clinical Trial (DIRECT-MT), Direct Endovascular Thrombectomy vs. Combined IVT and Endovascular Thrombectomy for Patients With Acute Large Vessel Occlusion in the Anterior Circulation (DEVT), and Direct Mechanical Thrombectomy in Acute LVO Stroke (SKIP). The main characteristics of these included RCTs were summarized in Tables 1, 2. All three trials were considered to have a low risk of bias (online-only Data Supplement), as assessed by the Cochrane Risk of Bias Tool. Among these three RCTs, a total of 1,092 individuals were enrolled, including 543 patients who were assigned to undergo MT alone (MT alone group) and 549 were assigned to receive combination therapy with intravenous alteplase and EVT (alteplase with EVT group). The distributions of the basic characteristics of the patients included in the analysis were similar across studies, including demographics and clinical characteristics (Table 2).

**Table 1:** Characteristics of studies included in meta-analysis.

**Trial characteristics**	**DIRECT-MT**	**DEVT**	**SKIP**
Inclusion criteria	1. Age of 18 years or older; 2. A clinical diagnosis of acute ischemic stroke and eligible for IVT and MT (within 4.5 hours after symptom onset, NIHSS ≥ 2); 3. Caused by a large vessel occlusion of the anterior circulation (intracranial segment of internal carotid artery, M1 segment of the middle cerebral artery, proximal M2 segment of the middle cerebral artery) confirmed by CTA; 4. CT or MRI ruling out intracranial hemorrhage; 5. Written informed consent.	1. Aged 18 years or older; 2. Presenting with acute ischemic stroke symptom within 4.5 hours and eligible for intravenous alteplase; 3. Occlusion of the intracranial internal carotid artery or the first segment of the middle cerebral artery confirmed by CT or MR angiography; 4. Randomization no later than 4 hours 15 minutes after stroke symptom onset. Time of stroke onset was defined as time last known well; 5. Informed consent obtained from patients or their legal representatives.	1. Age ≥18 and <86 years at the time of informed consent; 2. Clinical diagnosis of acute ischemic stroke with clinical symptoms and initial NIHSS ≥6; Modified Rankin scale score ≤ 2; 3. ICA or M1 occlusion on MRA or CTA; ASPECTS on initial DWI ≥5 or on initial CT ≥6; 4. Onset to randomization within 4 h from onset; 5. Written informed consent by patient or next of kin.
Exclusion criteria	1. Pre-stroke disability which interferes with the assessment of functional outcome at 90 days, i.e., mRS >2; 2. Any contra-indication for IVT, according to guidelines of the AHA, i.e.: (1) blood pressure > 185/110 mmHg; (2) blood glucose <2.7 or > 22.2 mmol/L; (3) cerebral infarction in the previous 6 weeks with residual neurological deficit or signs of recent infarction on neuro-imaging; (4) serious head trauma in the previous 3 months; (5) major surgery or serious trauma in the previous 2 weeks; (6) gastrointestinal or urinary tract hemorrhage in the previous 3 weeks; (7) previous intracerebral hemorrhage; (8) use of anticoagulant with INR exceeding 1.7; (9) known thrombocyte count <100 ×109/L; (10) treatment with direct thrombin or factor X inhibitors; (11) treatment with heparin (APTT exceeds the upper limit of normal value) in the previous 48 h.	1. CT or MR evidence of hemorrhage (the presence of micro-bleeds is allowed); 2. Contraindications of intravenous alteplase; 3. Premorbidity with a modified Rankin scale score of 0–2; 4. Currently in pregnant or lactating or serum beta HCG test is positive on admission; 5. Contraindication to radiographic contrast agents, nickel, titanium metals, or their alloys; 6. Arterial tortuosity and/or other arterial diseases that would prevent the device from reaching the target vessel; 7. Patients with a preexisting neurological or psychiatric disease that would confound the neurological functional evaluations; 8. Patients with occlusions in multiple vascular territories (e.g., bilateral anterior circulation, or anterior/posterior circulation); 9. CT or MR evidence of mass effect or intracranial tumor (except small meningioma); 10. CT or MR evidence of cerebral vasculitis; 11. CT or MR angiography evidence of intracranial arteriovenous malformations or aneurysms; 12. Any terminal illness with a life expectancy of <6 months; 13. Unlikely to be available for 90-day follow-up; 14. Current participation in another clinical trial.	1. Contraindication for contrast agent or endovascular therapy; 2. Contraindication for IVT ▪ Presence of severe renal disorder (patients undergoing dialysis can be included); 3. Pregnancy or possibility of pregnancy; 4. Unlikely to complete the study, such as due to progressive malignant tumor; 5. Judged incompatible with the study by the investigators.

*CTA, computed tomography angiography; IVT, intravenous thrombolysis; MT, mechanical thrombectomy; NIHSS, National Institutes of Health Stroke Scale; mRS: modified Rankin scale; HCG, human chorionic gonadotropin; ICA, internal carotid artery; DWI, diffusion weighted imaging*.

**Table 2:** Baseline patient characteristics among included randomized clinical trials.

**Characteristics**	**DIRECT-MT**		**DEVT**		**SKIP**	
	**Mechanical thrombectomy alone** **(*n* = 327)**	**Alteplase with endovascular thrombectomy** **(*n* = 329)**	**Mechanical thrombectomy alone** **(*n* = 116)**	**Alteplase with endovascular thrombectomy** **(*n* = 118)**	**Mechanical thrombectomy alone** **(*n* = 101)**	**Alteplase with endovascular thrombectomy** **(*n* = 103)**
Age, mean (SD) or median (IQR), y	69 (61–76)	69 (61–76)	70 (60-77)	70 (60-78)	74 (67-80)	76 (67-80)
Men, No. (%)	189 (57.8)	181 (55.0)	66 (56.9)	66 (55.9)	56 (55)	72 (70)
**Medical history**						
Hypertension, No. (%)	193 (59.0)	201 (61.1)	69 (59.5)	74 (62.7)	61 (60)	61 (59)
Atrial fibrillation, No. (%)	152 (46.5)	149 (45.3)	62 (53.5)	62 (52.5)	57 (56)	64 (62)
Diabetes, No. (%)	59 (18.0)	65 (19.8)	25 (21.6)	20 (17.0)	16 (16)	17 (17)
Ischemic stroke, No. (%)	43 (13.1)	47 (14.3)	14 (12.1)	19 (16.1)	12 (12)	14 (14)
**TOAST classification**						
Large artery (atherosclerosis), No. (%)	60 (18.3)	48 (14.6)	60 (51.7)	51 (43.2)	21 (21)	15 (15)
Cardioembolism, No. (%)	146 (44.6)	144 (43.8)	65 (56.0)	69 (58.5)	67 (66)	72 (70)
Other determined/undetermined etiology, No. (%)	121 (37.0)	137 (41.6)	19 (16.4)	21 (17.8)	13 (13)	16 (16)
**NIHSS score, median (IQR)**						
NIHSS score, median (IQR)	17 (12–21)	17 (14–22)	16 (12–20)	16 (13–20)	19 (13–23)	17 (12–22)
Baseline ASPECTS, median (IQR)	9 (7–10)	9 (7–10)	8 (7–9)	8 (7–9)	7 (6–9)	8 (6–9)
Systolic blood pressure, median (IQR), mm Hg	146 (130–163)	146 (131–161)	146 (129–165)	145 (128–168)	158 (132–172)	150 (134–171)
Glucose level, median (IQR), mmol/L or mean (SD), mg/dL	7.0 (5.8–8.6)	7.0 (5.9–8.8)	6.7 (5.7–8.1)	6.9 (5.9–8.9)	135 (48)	135 (52)
**Occlusion site**, ***n*** **(%)**						
Internal carotid artery	112/320 (35.0)	114/326 (35.0)	18/115 (15.5)	17/117 (14.4)	36 (36)	36 (35)
M1 MCA	161/320 (50.3)	178/326 (54.6)	95/115 (81.9)	99/117 (83.9)	54 (53)	47 (46)
M2 MCA	42/320 (13.1)	33/326 (10.1)	3/115 (2.6)	2/117 (1.7)	10 (10)	20 (19)

*IQR, interquartile range; TOAST, Trial of ORG 10172 in Acute Stroke Treatment; NIHSS, National Institutes of Health Stroke Scale; ASPECTS, Alberta Stroke Program Early CT Score; MCA, middle cerebral artery*.

### Primary Outcome

**Figure 1:**
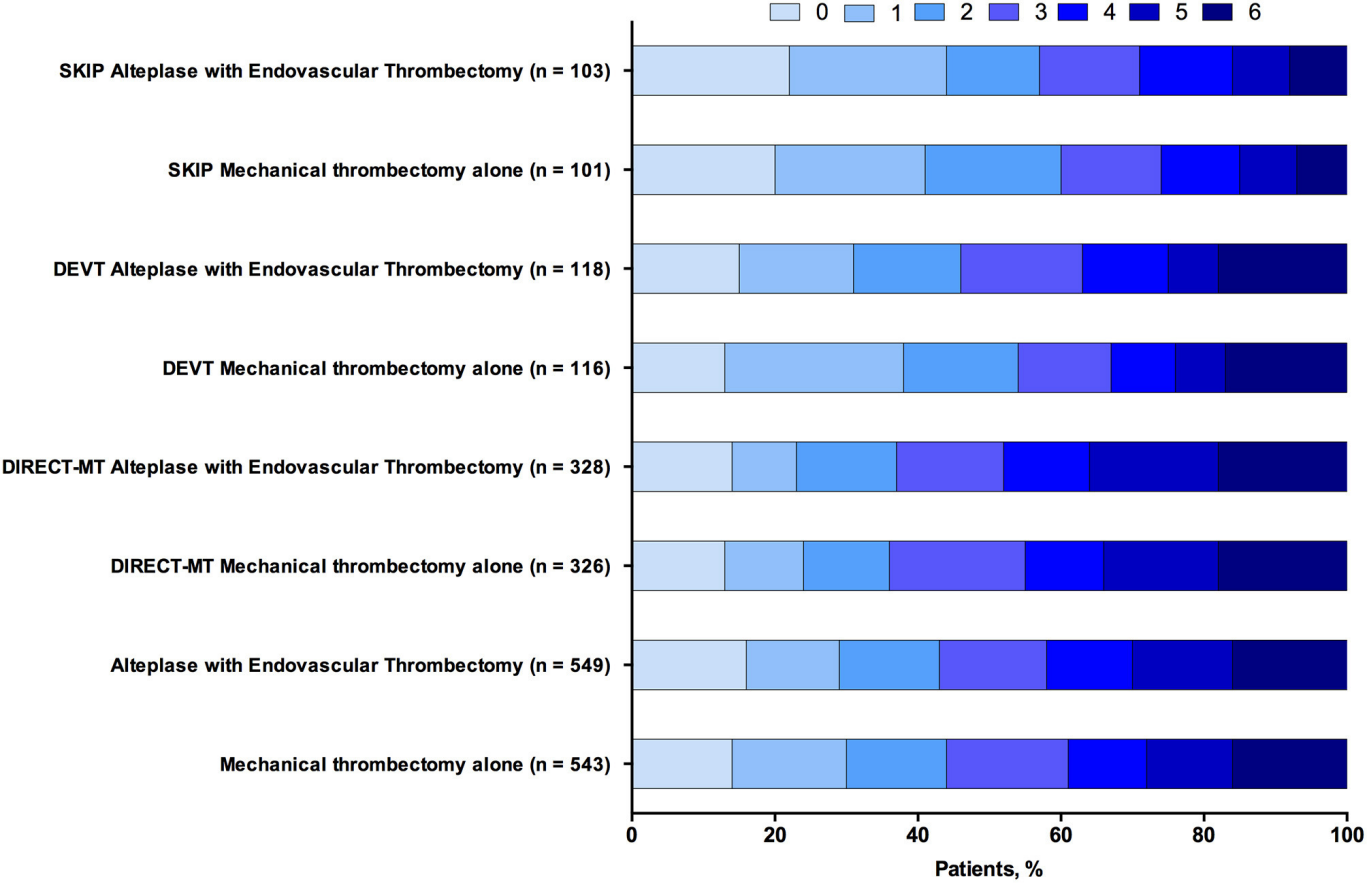
Functional outcome at 90-day follow-up of endovascular thrombectomy alone vs. alteplase with endovascular thrombectomy. The modified Rankin scale measures functional outcome on a seven-point ordinal scale: 0, no symptoms at all; 1, no significant disability despite symptoms; 2, slight disability; 3, moderate disability; 4, moderately severe disability; 5, severe disability; 6, death.

In the three trials and 1,092 patients with acute ischemic stroke that were included in the analysis of the primary outcome of 90-day functional independence (mRS score ≤ 2). The main analysis of the primary outcome showed no significantly different results in favor of the MT alone group (OR, 1.08 [95% CI, 0.85–1.38]; *P* = 0.054) (Table 3). The result of the score χ^2^ test to assess the proportional assumption was not significant (*P* = 0.573), which indicates that the proportional odds assumption is acceptable. The *I*^2^ value (variation in OR attributable to heterogeneity) was estimated as 0, which indicates no obvious heterogeneity was detected in the primary outcome. Moreover, Figure 1 shows a graphical summary of the seven scores of the mRS between both MT alone and alteplase with EVT groups at 90 days for the individual trials and pooled results.

**Table 3:** Distribution of 90-day modified Rankin scale scores.

**Modified rankin scale score**	**No. (%)**					
	**DIRECT-MT**		**DEVT**		**SKIP**	
	**Mechanical thrombectomy alone (*n* = 326)**	**Alteplase with endovascular thrombectomy (*n* = 328)**	**Mechanical thrombectomy alone (*n* = 116)**	**Alteplase with endovascular thrombectomy (*n* = 118)**	**Mechanical thrombectomy alone (*n* = 101)**	**Alteplase with endovascular thrombectomy (*n* = 103)**
0	43	45	15	18	20	23
1	37	29	29	19	21	23
2	39	47	19	18	19	13
3	63	48	15	20	14	14
4	36	38	10	14	11	13
5	50	59	8	8	8	8
6	58	62	20	21	8	9

**Table 4:** Summary of pooled analyses for primary and secondary outcomes.

**Outcomes**	**DIRECT-MT**		**DEVT**		**SKIP**		**Meta-analysis results**	
	**Mechanical thrombectomy alone (*n* = 327)**	**Alteplase with endovascular thrombectomy (*n* = 329)**	**Mechanical thrombectomy alone (*n* = 116)**	**Alteplase with endovascular thrombectomy (*n* = 118)**	**Mechanical thrombectomy alone (*n* = 101)**	**Alteplase with endovascular thrombectomy (*n* = 103)**	**OR (95% CI)**	***P*-value**
**Primary outcome**								
Functional independence	119 (36.4)	121 (36.8)	63 (54.3)	55 (46.6)	60 (59.4)	59 (57.3)	1.08 (0.85–1.38)	0.539
**Secondary outcomes**								
Excellent outcome	80 (24.5)	74 (22.5)	44 (37.9)	37 (31.4)	41 (40.6)	46 (44.7)	1.12 (0.85–1.47)	0.418
Successful reperfusion (TICI 2b-3), No. (%)	243/306 (79.4)	267/316 (84.5)	100 (88.5)	102 (87.2)	91 (90.1)	96 (93.2)	0.75 (0.54–1.05)	0.099
sICH, *n* (%)	14 (4.3)	20 (6.1)	10/115 (8.7)	12/115 (10.3)	8 (7.9)	12 (11.7)	0.72 (0.45–1.15)	0.171
Mortality at 90 days, *n* (%)	58 (17.7)	62 (18.8)	20 (17.2)	21 (17.8)	8 (7.9)	9 (8.7)	0.93 (0.68–1.29)	0.673

*OR, odds ratio; TICI, thrombolysis in cerebral infarction; sICH, symptomatic intracranial hemorrhage*.

### Secondary Outcomes

In the three trials and 1,092 patients that were included in the analysis of the excellent outcome (mRS score ≤ 1). An excellent outcome was observed in 144 of 543 patients (26.5%) in the MT alone group and 134 of 549 (24.4%) in the alteplase with EVT group (OR, 1.12 [95% CI, 0.85–1.47]; *P* = 0.418) (Table 3). Moreover, there was no significant difference in mortality rate at 90 days between MT and alteplase with EVT groups (OR, 0.93 [95% CI, 0.68–1.29]; *P* = 0.673) (Table 3). The percentage of patients with successful reperfusion (modified Thrombolysis in Cerebral InfarctionScore (mTICI) score, ≥2b) also showed no significant difference between two groups (OR, 0.75 [95% CI, 0.54–1.05]; *P* = 0.099) (Table 3). For safety outcome, the rate of symptomatic intracerebral hemorrhage in the two groups was 5.9% (32/542) vs. 8.0% (44/548) and did not differ significantly between the groups (OR, 0.72 [95% CI, 0.45–1.15]; *P* = 0.171) (Table 3).

## Discussion

In this meta-analysis, we comprehensively compared direct EVT vs. EVT with prior IVT for a large sample of acute ischemic stroke patients caused by a large vessel occlusion in the anterior circulation within 4.5 h from onset. We found that EVT with prior IVT does not appear to provide a functional outcome at 3 months over direct EVT for acute ischemic stroke patients who were eligible for treatment with both IVT and EVT. In addition, compared with direct EVT, the combination of IVT and EVT was non-inferior concerning early recanalization and reperfusion [TICI score 2b/3 after EVT or its equivalents], sICH, mortality at 90 days, 3-month excellent outcome (mRS 0–1).

Our findings are in contrast with the results of previous retrospective studies that reported worse functional outcomes in patients experiencing an acute ischemic stroke due to a large vessel occlusion who received general EVT alone compared with those who EVT with prior IVT (13–15). Previous meta-analysis studies also have examined differences between EVT with prior IVT vs. EVT alone, which have observed a trend toward higher rates of functional independence and successful recanalization among patients treated with IVT + EVT compared with patients treated only with EVT (5, 16, 17). In contrast to the aforementioned studies, a patient-level, pooled, *post hoc* analysis of the SWIFT and STAR studies revealed that treatment of patients experiencing an acute ischemic stroke due to a large vessel occlusion with IVT before EVT does not appear to provide a clinical benefit over EVT alone (4). However, there were important imbalances regarding inclusion criteria between groups in these studies, which make the data somewhat difficult to interpret. Most of the patients received EVT alone with contraindications for IVT treatment, including an extended period after known symptom onset, the unknown onset of stroke symptoms, or contraindications to IVT. Furthermore, to find randomized evidence to support or refute the role of IVT + EVT compared with EVT alone for patients with acute ischemic stroke, pooled analyses of randomized studies were conducted and attempted to resolve this issue, while the conclusions of these studies were also based on patients with contraindications to Tissue Plasminogen Activator (tPA), thus increasing the risk for bias and confounding. In consist with the results of our study, a meta-analysis conducted by Kaesmacher et al., which used only Recombinant Tissue Plasminogen Activator (rt-PA)–eligible patients did not find any benefit of EVT alone over EVT with prior intravenous alteplase (18). However, a common limitation of the aforementioned studies was that these meta-analyses pooled data mainly from retrospective cohort studies where the choice of EVT alone or EVT with prior intravenous alteplase for a given patient experiencing an acute ischemic stroke due to a large vessel occlusion was not randomized. Thus, the results of these meta-analyses may be confounded by indication and selection bias since all major guidelines recommend IVT in eligible patients before EVT (19).

Using IVT leading to favorable outcomes may associate with early recanalization for patients in the EVT with prior intravenous alteplase group. However, data from recent trials suggested that such early recanalization does not occur often. In the Multicenter Randomized Clinical Trial of Endovascular Treatment of Acute Ischemic Stroke in the Netherlands (MR CLEAN) and Endovascular Treatment for Small Core and Anterior Circulation Proximal Occlusion with Emphasis on Minimizing CT to Recanalization Times (ESCAPE) trials, only eight of 216 patients (3.7%) and eight of 165 patients (4.8%) randomized to MT had TICI 2b or 3 on the first angiography run, respectively (1, 20). The chance of early recanalization in response to IVT is associated with the location of the occlusion, with M2 or M3 occlusions responding effectively compared with distal ICA occlusions. In the DEVT trial, the middle cerebral artery M2 occlusions were excluded. Thereby, higher rates of successful reperfusion before thrombectomy were seen with combined intravenous alteplase and EVT in the DIRECT-MT and SKIP trials but not in the DEVT trial.

The benefit of recanalization after acute ischemic stroke is highly time-sensitive (21), thus the time delay due to the preparation of alteplase administration, which might be considered to be disadvantaged. A *post hoc* analysis of the MR CLEAN trial revealed that the median door-to-groin-puncture time was 11 min longer in non-transferred patients in the IVT + EVT group, which indicates that administration of IVT might contribute to a small delay in the start of EVT (22). However, this meta-analysis showed either no significant or no clinically relevant differences in most pre-specified time intervals. Although several time intervals were shorter in the direct EVT group, no significant differences were found in randomization to puncture time or arrival to arterial puncture between the treatment groups, and the mean time delay due to the preparation of alteplase administration was only about 3 min in SKIP study (16 min [IQR, 11–24] vs. 19 min [13–27], *P* = 0.38).

The results of this study are not sufficient to support clinical practice and paradigm shift toward direct EVT for patients with acute ischemic stroke from large-vessel occlusion. However, they support the hypothesis that EVT alone was non-inferior to combined IVT and EVT in these patients. Moreover, the results of this study are probably a consequence of the standardized workflow instituted in all three randomized clinical trials, which may not have been present in individual centers participating in previous non-randomized studies. Furthermore, strengths of the present meta-analysis include the conduction and report of the analysis according to the PRISMA.

However, a common limitation of these studies was that all three trials were conducted in East Asia, limited racial/ethnic diversity, thus increasing the risk for bias and confounding. Second, additional three randomized clinical trials (MR CLEAN-NO IV [ISRCTN80619088], SWIFT DIRECT [NCT03192332], and DIRECT-SAFE [NCT03494920]) to examine whether MT alone is non-inferior to combined IVT plus MT are ongoing. An updated meta-analysis may be needed in the future. Third, we were only able to get part of the data among the included trials. Some of the baseline characteristics were unavailable. Thus, we could not conduct some subgroup analysis, such as by baseline NIHSS score, occluded artery, and time to treatment. Despite these limitations, our study represented the best available pieces of evidence regarding EVT alone was non-inferior to combined IVT and EVT on the outcomes of patients with acute ischemic stroke with large-vessel occlusion in the anterior circulation.

## Conclusions

The pooled data from our meta-analysis of RCTs suggested that among patients with acute ischemic stroke in the anterior circulation within 4.5 h from the onset, EVT alone was non-inferior to combined IVT and EVT.

## Data Availability Statement

The original contributions presented in the study are included in the article/Supplementary Material, further inquiries can be directed to the corresponding author/s.

## Author Contributions

LL conceived the study. T-FW, G-CC, and JC collected the data and drafted the manuscript. H-SC, LL, and JC revised the manuscript and language. All authors contributed to the article and approved the submitted version.

## Funding

The work was supported by grants from the National Nature Science Foundation of China (No. 81901217).

## Conflict of Interest

The authors declare that the research was conducted in the absence of any commercial or financial relationships that could be construed as a potential conflict of interest.

## Publisher's Note

All claims expressed in this article are solely those of the authors and do not necessarily represent those of their affiliated organizations, or those of the publisher, the editors and the reviewers. Any product that may be evaluated in this article, or claim that may be made by its manufacturer, is not guaranteed or endorsed by the publisher.

## References

[1] Berkhemer OA Fransen PS Beumer D van den Berg LA Lingsma HF Yoo AJ . A randomized trial of intraarterial treatment for acute ischemic stroke. N Engl J Med. (2015) 372:11–20. 10.1056/NEJMoa1411587 25517348

[2] Saver JL Goyal M Bonafe A Diener HC Levy EI Pereira VM . Stent-retriever thrombectomy after intravenous t-PA vs. t-PA alone in stroke. N Engl J Med. (2015) 372:2285–95. 10.1056/NEJMoa1415061 25882376

[3] Goyal M Menon BK van Zwam WH Dippel DW Mitchell PJ Demchuk AM . Endovascular thrombectomy after large-vessel ischaemic stroke: a meta-analysis of individual patient data from five randomised trials. Lancet. (2016) 387:1723–31. 10.1016/S0140-6736(16)00163-X 26898852

[4] Coutinho JM Liebeskind DS Slater LA Nogueira RG Clark W Dávalos A . Combined intravenous thrombolysis and thrombectomy vs thrombectomy alone for acute ischemic stroke: a pooled analysis of the SWIFT and STAR studies. JAMA Neurol. (2017) 74:268–74. 10.1001/jamaneurol.2016.5374 28097310

[5] Mistry EA Mistry AM Nakawah MO Chitale RV James RF Volpi JJ . Mechanical thrombectomy outcomes with and without intravenous thrombolysis in stroke patients: a meta-analysis. Stroke. (2017) 48:2450–6. 10.1161/STROKEAHA.117.017320 28747462

[6] Yang P Zhang Y Zhang L Zhang Y Treurniet KM Chen W . Endovascular Thrombectomy with or without Intravenous Alteplase in Acute Stroke. N Engl J Med. (2020) 382:1981–93. 10.1056/NEJMoa2001123 32374959

[7] Zi W Qiu Z Li F Sang H Wu D Luo W . Effect of endovascular treatment alone vs intravenous alteplase plus endovascular treatment on functional independence in patients with acute ischemic stroke: the DEVT randomized clinical trial. JAMA. (2021) 325:234–43. 10.1001/jama.2020.23523 33464335 PMC7816099

[8] Suzuki K Matsumaru Y Takeuchi M Morimoto M Kanazawa R Takayama Y . Effect of mechanical thrombectomy without vs with intravenous thrombolysis on functional outcome among patients with acute ischemic stroke: the SKIP randomized clinical trial. JAMA. (2021) 325:244–53. 10.1001/jama.2020.23522 33464334 PMC7816103

[9] Moher D Liberati A Tetzlaff J Altman DG. Preferred reporting items for systematic reviews and meta-analyses: the PRISMA statement. BMJ. (2009) 339:b2535. 10.1136/bmj.b2535 19622551 PMC2714657

[10] Higgins JP Altman DG Gotzsche PC Juni P Moher D Oxman AD . The Cochrane Collaboration's tool for assessing risk of bias in randomised trials. BMJ. (2011) 343:d5928. 10.1136/bmj.d5928 22008217 PMC3196245

[11] Borenstein M Higgins JP. Meta-analysis and subgroups. Prev Sci. (2013) 14:134–43. 10.1007/s11121-013-0377-7 23479191

[12] Higgins JP Thompson SG Deeks JJ Altman DG. Measuring inconsistency in meta-analyses. BMJ. (2003) 327:557–60. 10.1136/bmj.327.7414.557 12958120 PMC192859

[13] Maingard J Shvarts Y Motyer R Thijs V Brennan P O'Hare A . Outcomes of endovascular thrombectomy with and without bridging thrombolysis for acute large vessel occlusion ischaemic stroke. Intern Med J. (2019) 49:345–51. 10.1111/imj.14069 30091271

[14] Ferrigno M Bricout N Leys D Estrade L Cordonnier C Personnic T . Intravenous recombinant tissue-type plasminogen activator: influence on outcome in anterior circulation ischemic stroke treated by mechanical thrombectomy. Stroke. (2018) 49:1377–85. 10.1161/STROKEAHA.118.020490 29748424

[15] Minnerup J Wersching H Teuber A Wellmann J Eyding J Weber R . Outcome after thrombectomy and intravenous thrombolysis in patients with acute ischemic stroke: a prospective observational study. Stroke. (2016) 47:1584–92. 10.1161/STROKEAHA.116.012619 27217508

[16] Fan L Zang L Liu X Wang J Qiu J Wang Y. Outcomes of mechanical thrombectomy with pre-intravenous thrombolysis: a systematic review and meta-analysis. J Neurol. (2020). 10.1007/s00415-020-09778-4 32140863

[17] Wang Y Wu X Zhu C Mossa-Basha M Malhotra A. Bridging thrombolysis achieved better outcomes than direct thrombectomy after large vessel occlusion: an updated meta-analysis. Stroke. (2021) 52:356–65. 10.1161/STROKEAHA.120.031477 33302795

[18] Kaesmacher J Mordasini P Arnold M López-Cancio E Cerdá N Boeckh-Behrens T . Direct mechanical thrombectomy in tPA-ineligible and -eligible patients versus the bridging approach: a meta-analysis. J Neurointerv Surg. (2019) 11:20–7. 10.1136/neurintsurg-2018-013834 29705773 PMC6327861

[19] Powers WJ Rabinstein AA Ackerson T Adeoye OM Bambakidis NC Becker K . 2018 Guidelines for the early management of patients with acute ischemic stroke: a guideline for healthcare professionals from the American Heart Association/American Stroke Association. Stroke. (2018) 49:e46–110. 10.1161/STR.0000000000000158 29367334

[20] Goyal M Demchuk AM Menon BK Eesa M Rempel JL Thornton J . Randomized assessment of rapid endovascular treatment of ischemic stroke. N Engl J Med. (2015) 372:1019–30. 10.1056/NEJMoa1414905 25671798

[21] Saver JL. Time is brain—quantified. Stroke. (2006) 37:263–6. 10.1161/01.STR.0000196957.55928.ab 16339467

[22] Chalos V LeCouffe NE Uyttenboogaart M Lingsma HF Mulder M Venema E . Endovascular treatment with or without prior intravenous alteplase for acute ischemic stroke. J Am Heart Assoc. (2019) 8:e011592. 10.1161/JAHA.118.011592 31140355 PMC6585366

