# Supplementary material for: Direct Endovascular Thrombectomy or With Prior Intravenous Thrombolysis for Acute Ischemic Stroke: A Meta-Analysis
Source: Front Neurol. 2021 Dec 13;12:752698. doi: 10.3389/fneur.2021.752698 (PMC8710447; doi:10.3389/fneur.2021.752698)
Supplement: Supplementary file 1 [file Table_1.DOCX]

**Direct Endovascular Thrombectomy or with Prior Intravenous Thrombolysis for Acute Ischemic Stroke: A Meta-analysis of Randomized Clinical Trials**

**Supplementary Online Content**

**Figure S1.** Flowchart of Literature Search and Study Selection

**Table S1.** Assessment of the Methodological Quality of Included Randomized Trials Using the Cochrane Collaboration’s Tool

**Figure S1.** Flowchart of Literature Search and Study Selection


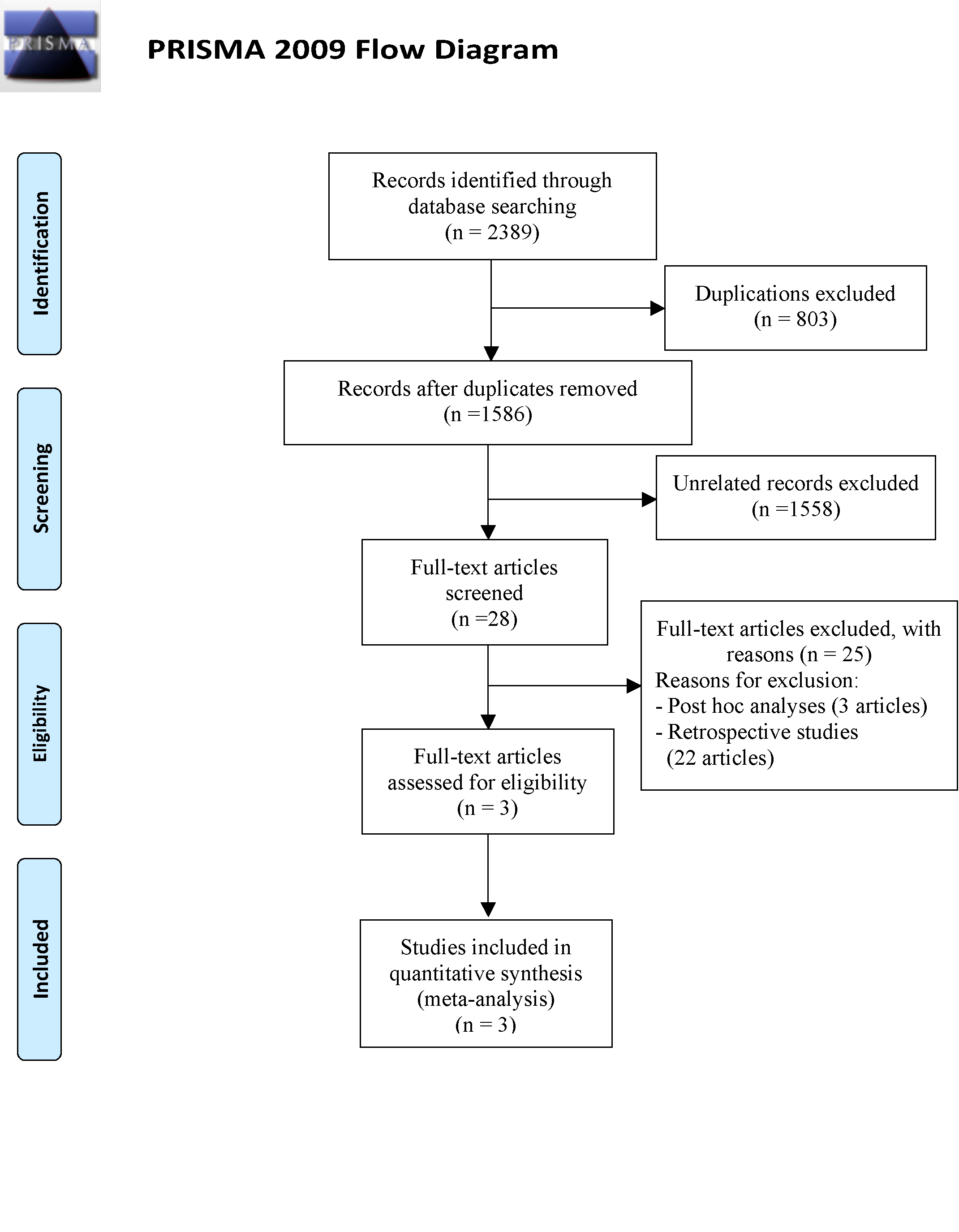


**Table S2.** Assessment of the Methodological Quality of Included Randomized Trials Using the Cochrane Collaboration’s Tool

| **Trials** | Sequence generation | Allocation concealment | Blinding of participants, personnel and outcome assessors | Incomplete outcome data | Selective outcome reporting | Other sources of bias |
| --- | --- | --- | --- | --- | --- | --- |
| DIRECT-MT | Low | Low | High | Low | Low | Low |
| DEVT | Low | Low | High | Low | Low | Low |
| SKIP | Low | Low | High | Low | Low | Low |
